# Regulatory T cell therapies: from patient data to biological insights

**DOI:** 10.3389/fimmu.2025.1675114

**Published:** 2025-10-31

**Authors:** Kameron B. Rodrigues, Peter J. Eggenhuizen, Rosa Bacchetta, Zinaida Good

**Affiliations:** ^1^ Division of Immunology and Rheumatology, Department of Medicine, Stanford University, Stanford, CA, United States; ^2^ Center for Biomedical Informatics Research, Department of Medicine, Stanford University, Stanford, CA, United States; ^3^ Center for Inflammatory Diseases, Department of Medicine, School of Clinical Sciences, Monash University, Clayton, VIC, Australia; ^4^ Division of Hematology, Oncology, Stem Cell Transplantation and Regenerative Medicine, Department of Pediatrics, Stanford University, Stanford, CA, United States; ^5^ Institute for Stem Cell Biology and Regenerative Medicine, Stanford University, Stanford, CA, United States; ^6^ Center for Definitive and Curative Medicine, Stanford University, Stanford, CA, United States; ^7^ Parker Institute for Cancer Immunotherapy, Stanford University, Stanford, CA, United States; ^8^ Weill Cancer Hub West, Stanford University, Stanford, CA, United States

**Keywords:** Treg, regulatory T cell, autoimmunity, transplantation, GvHD, T cell therapy, immune tolerance, immunomonitoring

## Abstract

Regulatory T cell (Treg) therapies are emerging as powerful tools for treating autoimmune and inflammatory diseases, preventing graft-versus-host disease (GvHD), and promoting organ transplant tolerance. Building on the identification of chimeric antigen receptor (CAR)-expressing Tregs as a correlate of poor patient outcomes in CD19-CAR T cell therapy, this review examines strategies for learning from clinical samples and data to improve Treg therapies. We highlight current and next-generation Treg modalities, including polyclonal, antigen-specific, converted, TCR-engineered, and CAR-engineered Tregs, provide a comprehensive overview of Treg clinical trials, and evaluate the evolving toolkit for *in vivo* Treg monitoring. Emphasis is placed on advanced immunomonitoring technologies, such as single-cell multi-omic profiling, epigenetic analysis, and spatial transcriptomics, which enable precise characterization of Treg persistence, function, and lineage stability. By integrating insights from adoptive T cell therapies and cutting-edge multi-omic platforms, this review outlines how Treg therapies can be optimized as “living drugs” capable of establishing immune tolerance across diverse clinical contexts.

## 1 Introduction

Regulatory T cells (Tregs) represent a specialized subset of CD4^+^ T lymphocytes crucial for maintaining immune homeostasis and preventing autoimmunity. Originally characterized by their high expression of CD25 (the IL-2 receptor α-chain) and the transcription factor FOXP3 (1–4), Tregs play an essential role in dampening excessive immune responses and promoting tolerance to self-antigens (5). Although detrimental in cancer, immunosuppressive functions have positioned Tregs as attractive candidates for cell-based therapies aimed at controlling unwanted immune reactions in autoimmune and inflammatory diseases, graft-versus-host disease (GvHD), and solid organ transplantation (6). Recent clinical observations from adoptive T cell therapy trials have underscored the potential for uncovering correlates of therapeutic outcomes and understanding the mechanism of failure in the context of cancer T cell therapies. Notably, the identification of chimeric antigen receptor (CAR)-expressing Tregs as negative correlates of patient outcomes in CD19-CAR T cell therapy for large B-cell lymphoma has provided insights on how engineered Tregs can be monitored in clinical settings and provided evidence for function of engineered Tregs in humans (7, 8). In this review, we examine the evolving landscape of clinical trials for Treg cell therapies, from non-engineered polyclonal Tregs to antigen-specific, T cell receptor (TCR)-engineered, and CAR-engineered Tregs. We discuss cutting-edge technologies for tracking and characterizing Tregs in patients and highlight operational considerations for maximizing insights from clinical trials. By drawing lessons from other adoptive transfer approaches, we aim to provide a framework for optimizing Treg therapies and expanding their clinical applications.

## 2 Treg cell therapies in the clinic

### 2.1 Polyclonal Tregs

**Figure 1:**
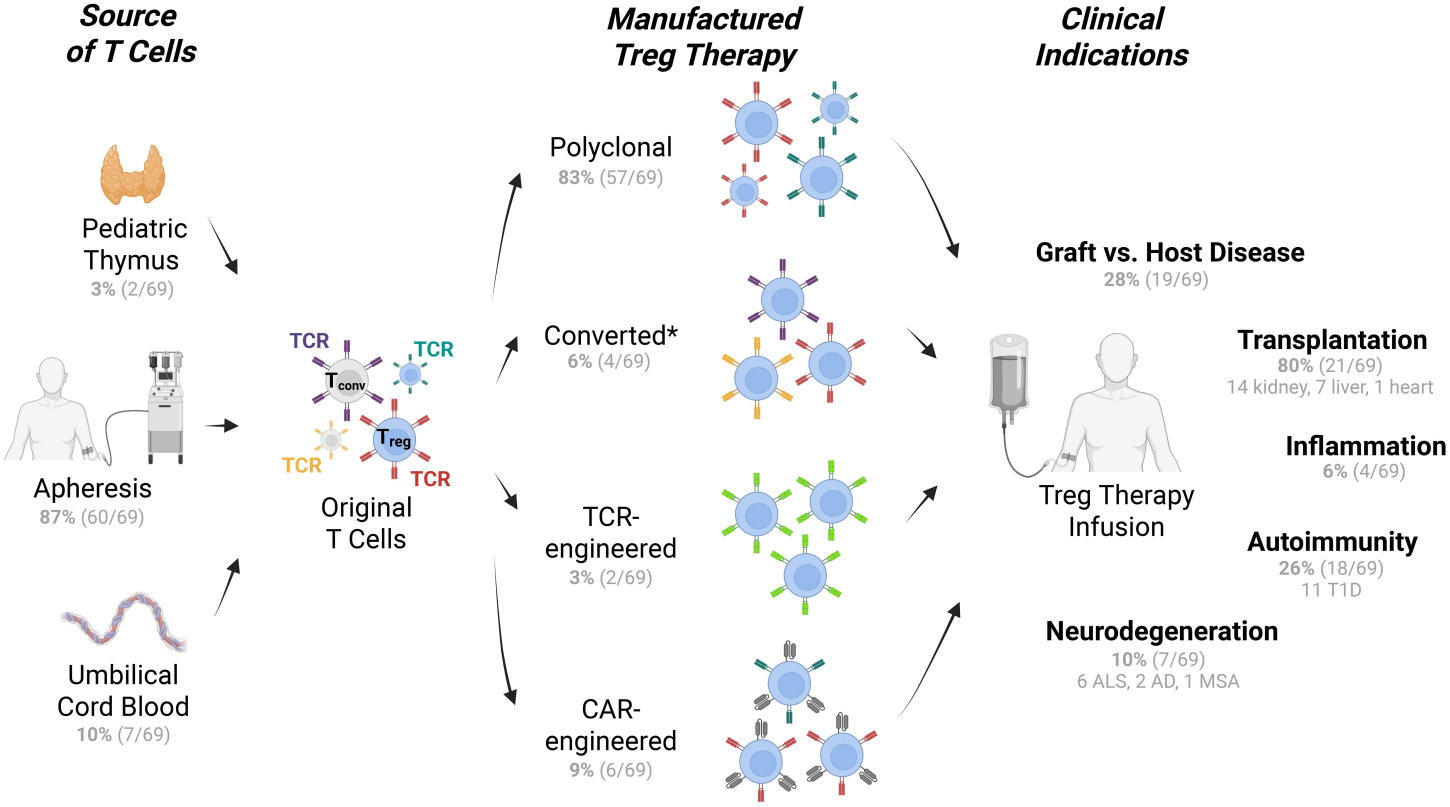
Types of Treg therapies, T cell sources, and indications in clinical trials. Treg therapies from multiple sources of T cells (*left*) are manufactured via multiple approaches (*middle*) for multiple applications under evaluation in clinical trials (*right*). Tconv cells are represented with grey cytoplasm and nucleus, while Tregs are represented in blue. Colored receptors denote TCR or CAR where relevant. The number of clinical trials are listed in parentheses. See Treg therapy clinical trial details in **Supplementary Table S1**. *Converted Treg products may originate from CD4^+^ T conventional cells. Trials that were withdrawn, terminated, or suspended were excluded. T1D, type 1 diabetes; ALS, amyotrophic lateral sclerosis; AD, Alzheimer's disease; MSA, multiple system atrophy (108).

The earliest clinical applications of Treg therapy employed non-engineered, polyclonal Tregs isolated from peripheral blood (9) (**Figure 1**). These approaches typically involved isolation of CD4^+^CD127^low^ T cells through fluorescence-activated cell sorting (FACS) and/or magnetic bead-based methods (often CliniMACS Plus System, Miltenyi Biotec, for CD25^+^ selection), followed by cryopreservation or direct administration (10–12). The CliniMACS bead enrichment approach, while practical, often results in around 80% Tregs mixed with other cell types (13–16) (NCT02371434, NCT02385019). Given how rare Tregs are in peripheral blood — comprising only 5-10% of CD4^+^ T cells (17, 18) (2-8% in our hands) — the field sought for additional clinical sources of Tregs with goals to improve purity. *Ex vivo* expansion methods were developed and implemented in clinical trials (19), specifically with anti-CD3/CD28 stimulation in the presence of high-dose interleukin-2 (IL-2) alone (20, 21), or with rapamycin — an inhibitor of mammalian target of rapamycin (mTOR) — resulting in improved Treg purity of about 90% (14, 22). Other sources of Tregs are also implemented in clinical trials, such as cryopreserved umbilical cord blood (NCT05027815, NCT05349591) or discarded thymus tissue from pediatric heart transplants (NCT04924491, NCT06052436). Polyclonal Tregs have shown promising safety profiles in allogeneic settings such as GvHD prevention and solid organ transplantation (22, 23), and also in autologous settings such as type 1 diabetes (T1D) (24). One important example of polyclonal non-engineered Treg therapy is Orca-T, where allogeneic (graft-matched) Tregs were freshly administered at a 1:1 ratio with T conventional cells (Tconv) along with CD34^+^ hematopoietic stem cells to prevent GvHD (10), leading to positive phase 2 trial results (25, 26).

Antigen-specific Tregs can be enriched from purified polyclonal Tregs through *ex vivo* expansion. In the context of allogeneic setting, host Tregs can be isolated then exposed to donor cells *ex vivo* to expand donor-alloantigen reactive Tregs. This approach has been used in clinical trials to prevent transplantation rejection for either kidney (NCT02091232, NCT02244801) (15, 16) or liver (NCT02188719, NCT02474199, NCT03577431, NCT03654040). Enriched antigen-specific Tregs have also been applied in autologous settings. In a trial for Alzheimer’s disease (AD), Tregs were expanded *ex vivo* in the presence of amyloid beta antigen to enrich for amyloid beta reactive Tregs (27). An interesting extension of this approach is ‘CRANE’ technology from Cellenkos that expands Tregs from cord blood while enriching for a specific population that has high levels of specific homing receptors. For example, Tregs expressing integrin protein CD11a, in the case of CK0803 for trial NCT05695521, have been used to target the CXCR3/CXCL10 axis with the aim of engaging the inflamed microglia in patients with amyotrophic lateral sclerosis (ALS). Additionally, CD49d is targeted in CK0802 for trial NCT04468971 (28).

Although approaches for *ex vivo* polyclonal Treg expansion faced several limitations, the early clinical experiences with polyclonal Tregs provided valuable insights into dosing, safety, and monitoring strategies. Polyclonal Treg trials have advanced into late-stage trials despite the limitations of restricted antigen specificity, possible *in vivo* instability, variable purity, and limited persistence. In summary, the trials for polyclonal Tregs have paved the way for next-generation Treg therapies by expanding Treg sources, improving cell isolation and expansion technologies, establishing the importance of antigen specificity, and providing initial evidence of clinical benefit in both autologous and allogeneic settings.

### 2.2 Converted Tregs

By 2025, approaches of polyclonal T cell products included the reprogramming of conventional T cells (Tconv) to acquire regulatory function, generating induced Treg (iTreg) or converted Treg cells (**Figure 1**). Generally, iTregs are produced in clinical trials by culturing CD4^+^CD25^–^ T cells with IL-2, rapamycin, transforming growth factor β (TGF-β), and anti-CD3 monoclonal antibody-loaded artificial antigen-presenting cells to generate FOXP3^+^ iTregs with potent suppressive function (NCT01634217 for GvHD) (29). A similar protocol leveraging rapamycin has been used to reprogram Tconv cells in the context of ALS and COVID-19 related acute respiratory distress syndrome. For example, NCT06169176, NCT04220190, and NCT04482699 utilize Rapa-501, a two-step, 7-day culture process. First, T cells are de-differentiated using rapamycin with media starvation, which drives T cells towards a T stem cell memory phenotype. The final step is to re-differentiate the T cells into Treg and Th2 programs with IL-2, IL-4, and TGF-β. As of 2025, NCT04220190 which used this rapamycin reprograming approach was the most progressed clinical trial of the converted Treg class and is in phase 3 for treatment of ALS. Cell engineering approaches for converted Treg products have begun clinical trials, where high FOXP3 expression is induced by lentiviral transduction of CD4^+^ T cells, together with a surface marker gene tNGFR. This autologous converted Treg-like cell product (CD4^LVFOXP3^) is administered to patients who genetically lack functional Tregs in a first-in-human trial for conditions including immune dysregulation, polyendocrinopathy, enteropathy, and X-linked (IPEX) syndrome (NCT05241444) (30). In a similar gene-transfer approach, high IL-10 production is induced by lentiviral transduction into CD4^+^ T cells (CD4^LV-IL10^) to produce allogenic type 1 regulatory T cells (Tr1 cells) for “off-the shelf” GvHD and inflammatory bowel disease (IBD) treatment in clinical trials led by Tr1X Bio (31, 32). Type 1 Tregs are important for peripheral tolerance, with suppressive functions mediated by IL-10, TGF-β, and CTLA4, independent of FOXP3 (33, 34). These approaches, which still produce polyclonal Treg-like cells, differ from expanded polyclonal Tregs in that they do not enrich for Tregs prior to differentiation/expansion, and rather utilize all CD4^+^ T cells as the starting material, overcoming the issues of reaching sufficient Treg number and purity. Converted Tregs are particularly valuable in settings where functional Tregs cannot be obtained in sufficient numbers as a starting material (*e.g.* IPEX), or where inflammation is naturally controlled by iTreg or Tr1 cells (*e.g.* IBD), with additional settings under investigation.

### 2.3 TCR-engineered Tregs

T cell receptor (TCR) engineering of Tregs renders them antigen-specific for a particular disease target and is an emerging approach yet to fully transition to the clinic (6). Preclinical models of transplantation tolerance demonstrate that TCR-engineered Tregs exhibit enhanced potency compared to polyclonal Treg populations and can mediate “linked suppression” of responses against other antigens present in the same microenvironment (35–37). To enhance antigen specificity and potentially improve therapeutic efficacy, the field is developing TCR-engineered Tregs. By introducing TCRs specific for relevant disease antigens (*e.g.*, alloantigens in transplantation or self-antigens in autoimmunity), TCR-engineered Tregs are expected to exert targeted immunosuppression at pathogenic sites (**Figure 1**). In the context of autoimmunity, preclinical models of TCR-engineered Tregs include the following targets: (i) myelin basic protein in multiple sclerosis (38), (ii) Smith autoantigen in lupus nephritis (39), (iii) factor VIII in hemophilia A (40), (iv) type IV collagen in anti-glomerular basement membrane disease (41), and (v) glutamic acid decarboxylase in type 1 diabetes (42). These studies demonstrated the potential of TCR-engineered Tregs in restoring immune tolerance. Key potential advantages of TCR-engineered Tregs, if successful, would include improved localization reachable with lower cell doses, enhanced persistence, superior antigen specificity, and reduced risk of undesired immunosuppression via lowered bystander suppression. However, challenges remain in selecting optimal target antigens, which are still largely unknown for the majority of autoimmune diseases (43), managing potential off-target effects and genotoxicity, and ensuring reliable and safe manufacturing of these more complex cellular products. As of late-2025, only two clinical trials existed for TCR-engineered Tregs. Abata therapeutics engineered autologous Tregs to express a TCR that specifically recognizes immunogenic myelin fragments in the CNS (ABA-101, NCT06566261). In late 2025 a phase 1 clinical trial begun for GENTI-122, a converted Treg product from Gentibio to treat T1D where CD4^+^ T cells are engineered to express FOXP3, a chemically inducible signaling complex (CISC) that provides IL-2 signaling support in response to rapamycin, and IGRP305-TCR that recognizes the pancreatic islet-specific glucose-6-phosphatase catalytic subunit–related protein (IGRP) peptide (NCT06919354) (44). More work is needed for progressing preclinical results of TCR-engineered Tregs into clinical trials, whereas the first TCR-engineered Treg trials will provide crucial data for future product optimization.

### 2.4 CAR-engineered Tregs

Chimeric antigen receptor (CAR) technology, which has revolutionized cancer immunotherapy (45), has been adapted to engineer Tregs with enhanced specificity and function (6). CAR Tregs express synthetic receptors that recognize cell surface antigens independent of MHC presentation, combining the specificity of an antibody with intracellular signaling and leading to Treg activation and regulatory function. Initial preclinical studies demonstrated that CAR Tregs targeting HLA-A2, factor VIII, or myelin oligodendrocyte glycoprotein (MOG) could suppress alloimmunity, autoimmunity against factor VIII in hemophilia, or experimental autoimmune encephalomyelitis, respectively (46–48). The first clinical trials of CAR Tregs are now underway, including a phase 1/2a trial of HLA-A2-specific CAR Tregs for prevention of kidney transplant rejection (Sangamo Therapeutics, NCT04817774) (49, 50) or liver transplant rejection (Quell Therapeutics, NCT05234190), citrullinated vimentin-specific CAR Tregs for rheumatoid arthritis (Sonoma Therapeutics, NCT06201416) (51) and hidradenitis suppurativa (NCT06361836), and CD6-specific CAR Tregs for GvHD (NCT05993611) (**Figure 1**). Although not yet in clinical trials, Tr1X Bio is developing TRX319, an allogeneic polyclonal CAR Treg therapy for the treatment of multiple B cell mediated autoimmune diseases. The finding that CD19-CAR Tregs correlate with poor outcomes in cancer immunotherapy further underscores the potential of CAR Treg therapies for *in vivo* suppression in clinical settings (7, 8). CAR Treg approaches offer several potential advantages, including MHC-independent recognition, tunable signaling domains, and diverse targeting options. However, the complexity of CAR Treg biology presents unique challenges, such as balancing activation and stability, preventing exhaustion or plasticity due to CAR tonic signaling, and addressing manufacturing considerations.

Leveraging our recently published list of Treg therapy clinical trials (52), we have retrieved data for each interventional trial and presented results in **Supplementary Table S1**, as summarized in **Figure 1**. Polyclonal Treg approaches are the most established, representing 83% of trials as of late 2025. Converted Tregs are emerging (representing 6% of all Treg trials). Among engineered Treg cell therapies, CAR Treg approaches are more mature than TCR-engineered Tregs (representing 9% *vs.* 3% of all Treg trials, respectively). It remains to be determined whether other forms of engineered or “modified” Tregs can be exploited in the clinic, for example to leverage Treg metabolism or enhance inflammatory activity in cancer settings (53–56).

## 3 Technologies for Treg immunomonitoring in clinical trials

**Figure 2:**
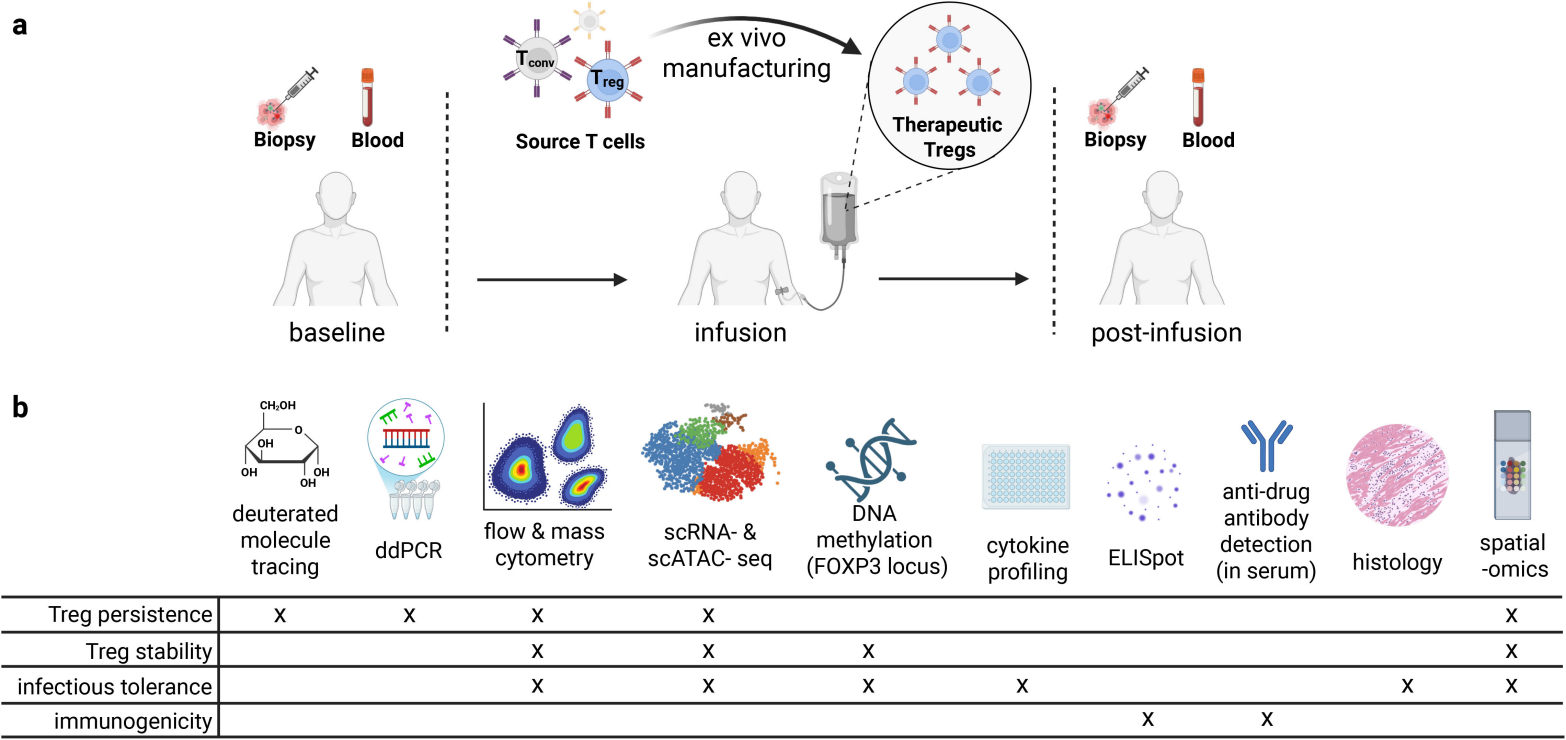
Experimental approaches for immune monitoring of Treg cell therapies in clinical trials. **(a)** Timepoints and sample types that can be valuable for correlative studies in clinical trials. **(b)** Assays and specific insights into properties of Treg cell therapies that can be obtained from relevant clinical samples (109).

Comprehensive monitoring of Treg therapies requires sophisticated technologies that can track cell persistence, phenotype, stability, immune rejection, tissue distribution, and function over time. In this process, it is also critical to monitor disease state, potential for infectious tolerance, and the overall immune state in the relevant tissues and blood. Several complementary approaches have emerged as essential tools for understanding Treg behavior *in vivo* (**Figure 2**).

### 3.1 Treg tracking methods

Historically, Jeffrey Bluestone’s group pioneered the first approach to tracking Tregs *in vivo*. The technique involves labeling Tregs with deuterium (²H) prior to infusion to enable long-term tracking of cell persistence and proliferation. By culturing Tregs in deuterated glucose (57) or media containing deuterated water during *ex vivo* expansion, Tregs incorporate the stable isotope into newly synthesized DNA and proteins (44, 58). After transfer, deuterium-labeled Tregs can be detected in blood and tissue samples through mass spectrometry, allowing assessment of persistence, proliferation, and tissue distribution. A phase I trial of deuterium-labelled polyclonal Tregs for type 1 diabetes showed safety and Treg detection for up to one year post-transfer (22). This technique provides unique insights into Treg kinetics *in vivo* without genetic manipulation, offering advantages for clinical studies where genetic tracking methods are not feasible.

Genetic tracking of engineered Tregs in clinical trials can be achieved by encoding an inert and non-immunogenic human cell surface transgenic protein that is detectable by flow cytometry (59) and immunohistochemistry. One example is truncated epidermal growth factor receptor (EGFRt), which can be detected by the antibody cetuximab that is available with good manufacturing practices (GMP) certification (60). EGFRt is utilized in CD19-CAR T cell trials (NCT05625594). It is also often used in pre-clinical testing of CAR Tregs (61). In addition to being an engineered Treg tracking tool, EGFRt can enable enrichment of engineered cells pre-transfer, as well as function as a ‘kill switch’ in case of toxicity or malignancy through cetuximab-mediated *in vivo* elimination. Another example of cell surface transgenic protein for tracking Tregs is truncated nerve growth factor receptor (tNGFR), also known as LNGFR or CD271, and utilized for GMP-compatible pre-transfer enrichment, post-transfer tracking, and quantification of engineered Tregs (30). tNGFR-transduced cells were shown to be safe (62) and were generated in the phase I clinical trial of CD4^LVFOXP3^ cells for IPEX (NCT05241444; see above). Alternatively, antibodies can be used to detect a functional engineered Treg protein, such as CAR or TCR (7). For example, CAR idiotype antibodies are specific to the scFv binding pocket of the CAR construct (59). Antibody-mediated detection of genetic Treg markers can be combined with single-cell technologies through CITE-seq (63) or spatial technologies, such as immunohistochemistry (IHC) (59) or CODEX (64), although CAR idiotype antibodies often have excessive background signal in spatial applications. DNA or RNA transcripts encoding engineered proteins remain detectable and can be traced to identify infused Tregs using quantitative real-time PCR, highly sensitive digital droplet PCR (ddPCR) (65, 66), single-cell sequencing, or spatial transcriptomics. Overall, engineered cell tracking technologies provide data on persistence and biodistribution of engineered Tregs in humans.

### 3.2 Assessing Treg phenotype, stability, and function

#### 3.2.1 Flow cytometry and mass cytometry

Flow cytometry remains the cornerstone of Treg identification and characterization in clinical samples. Conventional panels typically include markers such as CD4, CD25, CD127, and FOXP3, along with activation markers (*e.g.* CD39), homing receptors (*e.g.* CCR4), and functional markers (*e.g.* Ki-67) (67). Spectral flow cytometry panels enable practical quantification of 30–40 markers. Mass cytometry (CyTOF) extends this capability by enabling practical detection of 40–50 parameters using metal-tagged antibodies, allowing more comprehensive phenotyping with minimal spectral overlap (7, 68–70). This approach is generally applied to batched cryopreserved samples, revealing previously unappreciated heterogeneity within the Treg compartment and distinct Treg subpopulations associated with clinical outcomes (71). Key considerations for flow-based monitoring include thoughtful antibody panel development and validation, standardization of staining procedure (*e.g.* Using lyophilized, pre-mixed antibody panels formatted as single-bead aliquots), and including batch controls that express all markers up to maximum level in the test samples. These methods can provide critical information on Treg persistence and stability, functional and homing marker assessment, trafficking patterns, and the overall state of the immune system.

#### 3.2.2 Single-cell sequencing

Single-cell RNA-sequencing (scRNA-seq) has enabled deep characterization of the *in vivo* cellular heterogeneity and proven crucial for tracking Tregs during reconstitution post-stem cell transplantation (72). The comprehensive transcriptional profiles of individual cells provided by scRNA-seq reveals functional states, activation status, and potential loss of phenotypic and functional stability that may be missed by protein-based methods. In the context of Treg therapies, scRNA-seq is useful in identifying transcriptional signatures and pathways associated with therapeutic efficacy or toxicity, tracking clonal dynamics of transferred Tregs through integration with single-cell TCR sequencing (scTCR-seq) (73), detecting lineage instability through expression of non-Treg lineage genes, incorporating expression of key proteins through CITE-seq (63), and mapping interactions between Tregs and other immune or tissue cells through interactome analyses (74, 75).

Assay for transposase-accessible chromatin using sequencing (ATAC-seq) provides insights into the epigenetic landscape of cells by quantifying regions of open chromatin (76). When applied to Tregs, this technique reveals regulatory elements controlling Treg identity and function, including those associated with FOXP3 expression and stability (77, 78). Single-cell ATAC-seq (scATAC-seq) can identify epigenetic changes occurring in Treg subpopulations during therapy, potentially predicting functional alterations before they become apparent at the transcriptional or protein level (79, 80). Further, scATAC-seq can assess the extent that engineered Tregs recapitulate natural Tregs epigenetically (including at the FOXP3 locus), providing information on cell stability, enhancer activity, and the extent that Tregs are ‘primed’ for future cell states. Integration of scATAC-seq with scRNA-seq data through multi-omic approaches (81–84) enables trajectory inferencing (85, 86), while providing a more complete picture of the Treg cellular states and kinetics.

The main limitations of single-cell sequencing technologies for Treg clinical trials are the cost and limited cell numbers. Thus, correlative studies often leverage fluorescence-activated cell sorting (FACS) to enrich a population of interest – such as infused Tregs from blood – prior to scRNA-seq or scATAC-seq. Costs can be further reduced through selecting the most informative samples, barcoding and pooling samples in batched analyses, and leveraging rapidly evolving technologies (*e.g.* 10x Genomics GEM-X, BD Rhapsody, Parse Evecode, Illumina Single Cell) and kits for single-cell sequencing (*e.g.* 48-sample kit is more cost effective than a 16-sample kit).

#### 3.2.3 Spatial omics

Spatial omics technologies build on the original spatial analysis methods, including hematoxylin and eosin (H&E) stain, IHC, and immunofluorescence. Single-cell spatial transcriptomic tools, including Nanostring CosMx, 10x Genomics Xenium, and Vizgen MERSCOPE, utilize probes to detect a preset panel of up to 6,000 genes and support custom probes for engineered proteins, such as CAR or TCR. Spatial proteomics technologies, including MIBI (87) and CODEX (64, 88), can be used instead of or in parallel with spatial transcriptomics methods to learn insights from the relevant tissue biopsies. Already applied in studies on Treg therapy for kidney transplantation (89–92), spatial omics methods could be essential to comprehensively profile the immune state within the relevant tissue biopsies in Treg trials, detect Tregs in tissues and define their phenotype, and examine Treg-rich organized lymphoid structures (TOLS) (93), if present. In addition to assessing Treg persistence, phenotype, and microenvironment, spatial transcriptomics can define spatial cell-cell communication (94, 95). Important advantages of spatial omic technologies are spatial information and more accurate cell proportions when compared to single-cell analyses of dissociated tissues. Limitations of spatial omics include lower precision in cell type separation due to imperfect cell segmentation and spillover effects, higher background in spatial proteomics compared to flow cytometry, and higher dropout in spatial transcriptomics compared to single-cell sequencing. Constructing tissue microarrays (TMAs) from serial tissue biopsies can reduce costs of spatial omics analyses.

#### 3.2.4 TSDR demethylation

The biological instability of Tregs represents a concern for Treg cell therapies, as infused cells could lose their identity when exposed to inflammatory environments *in vivo*. This instability manifests as FOXP3 downregulation, phenotypic conversion, proinflammatory cytokine production, and unpredictable therapeutic performance (67). While single-cell technologies can assess Treg phenotype for inference of stability, DNA methylation information is considered gold standard. Treg identity and stability are closely linked to demethylation of specific regulatory regions of the *FOXP3* locus, particularly the Treg-specific demethylated region (TSDR) (96–99). Quantitative analysis of TSDR demethylation serves as a reliable measure of *bona fide* Tregs and can be used to track the stability of transferred Treg cell products over time.

#### 3.2.5 Treg functional assays

Assessing function of therapeutic Tregs in clinical samples remains an active area of methodological development. In addition to antigen-specific suppression, Tregs can induce bystander suppression to antigens that are distinct from their original antigenic specificity (100–103). Infectious tolerance is a phenomenon that could occur in the context of Treg therapies where Tregs induce tolerance in Tconv and other immune cells, effectively ‘spreading’ their regulatory function beyond their direct or bystander suppressive effects and potentially lasting even if the therapeutic Tregs wane (104, 105). Although *in vitro* suppression or antigen-specific suppression assays can provide evidence of Treg function in blood samples, the quality of clinical samples collected, stored, and transported over years may not always be sufficient for functional assays. Pathway activity or proliferation markers of therapeutic Tregs based on flow cytometry or scRNA-seq analysis can provide evidence of function (7). Further, measuring changes in regulatory plasma cytokines (*e.g.* IL-10, TGF-β) versus inflammatory cytokines can provide evidence of function and indirect evidence of infectious tolerance. Infectious tolerance and bystander suppression can be assessed using flow cytometry, single-cell sequencing, TSDR demethylation, and spatial omics analysis of tolerogenic features and reduction in inflammatory features among non-therapeutic cells, respectively (*e.g.* increase in FOXP3^+^Helios^–^ T cells may indicate *de novo* Treg induction). Finally, examining relevant tissue histology for a reduction in disease-specific features, tissue structures associated with tolerance (*e.g.* TOLS), and tolerogenic state of non-therapeutic cells using a combination of H&E, IHC, and spatial omics methods can provide vital information on therapeutic Treg suppression and infectious tolerance *in situ*. Clinical efficacy is ultimately the most important metric of therapeutic Treg function that is assessed through disease-specific metrics.

### 3.3 Monitoring Treg rejection

The immune system may recognize foreign antigens in allogeneic Tregs or in genetically modified Tregs, such as scFv in CAR or junction sequences in TCR, leading humoral (antibody-mediated) and cellular (T cell-mediated) rejection of the therapeutic Treg cells (106). Humoral rejection can be measured by ELISA of serum samples to detect anti-drug antibodies targeting the engineered protein. Cellular rejection can be tested in patient-derived peripheral blood mononuclear cells (PBMCs) (*e.g.* against peptides spanning the engineered protein) via IFN-γ production by ELISpot. Monitoring Treg rejection can provide valuable information when therapeutic Tregs do not persist.

## 4 Operational considerations and future directions

### 4.1 Maximizing insights from Treg correlative studies

To maximize biological insights from Treg clinical trials, we recommend a standardized yet flexible approach to sample collection and correlative assays (**Figure 2**). Longitudinal peripheral blood samples should be collected at baseline (ideally at the time of apheresis, if applicable), immediate (days 1-2), early (days 7-14), mid (weeks 4-8), and late (months 3-6) post-infusion; precise timing would be driven by the biology of disease and Treg therapy. At each timepoint, PBMCs, plasma, and serum should be processed and cryopreserved. Tissue biopsies — if clinically justified — should be collected at baseline and at matched post-infusion timepoints (*e.g.* 4–8 weeks and 3–6 months). Treg infusion products and pre-manufacture cell products should also be banked. We recommend spectral flow cytometry or mass cytometry analysis of all batched cryopreserved PBMCs, pre-manufacture cells, and Treg infusion products to assess Treg persistence, stability, and function and examine correlates of patient outcomes. Single-cell RNA-seq and paired scTCR-seq should ideally be performed on pre-manufacture cells, Treg infusion products, and FACS-enriched blood CD4^+^ T cells that are positive for a genetically encoded surface marker, if available (*e.g.* EGFRt, tNGFR, CAR). When performed at high-quality timepoints, these approaches enable deep profiling of infused Treg cell state and lineage stability. *FOXP3* TSDR demethylation assessed by bisulfite sequencing can provide gold standard information on Treg lineage stability and potential infectious tolerance. In tissue, immunohistochemistry on full slides and spatial transcriptomics on TMAs should be used to define Treg localization, phenotype, and tissue microenvironment. For engineered Tregs, ddPCR should assess persistence, whereas tracking non-engineered Tregs is limited to deuterium labeling and may be difficult to implement. Cytokine profiling (*e.g.* Luminex) of plasma and anti-drug antibody ELISA of serum provide functional and rejection data, respectively. When rejection is suspected, ELISpot for T cell responses against engineered domains is recommended. These harmonized protocols should be combined with monitoring relevant disease biomarkers to enhance biological insights across Treg therapy trials.

### 4.2 Future landscape of Treg cell therapy

The field of Treg cell therapy stands at an inflection point, with fundamental insights from preclinical studies and lessons from early clinical experiences converging to guide next-generation approaches (6). Future Treg cell therapies will likely be shaped by several emerging trends: engineered antigen specificity, allogeneic approaches for off-the-shelf availability, induced/converted Tregs to overcome natural Treg limitations, and controlled expansion *in vivo* to enhance persistence of therapeutically relevant cells. By learning from both successes and challenges of adoptive T cell therapies (107), the field can accelerate the development of Treg-based approaches that harness their full potential as ‘living drugs’ using cutting-edge technologies for engineering and monitoring Tregs, coupled with thoughtful trial design and data analysis strategies. With at least 69 Treg clinical trials across autoimmune and inflammatory diseases and transplantation as of 2025 (**Supplementary Table S1**), Treg cell therapies have demonstrated their potential for precise immune regulation that could transform treatment paradigms.

## Supplementary material

Supplementary Table 1 Treg therapy clinical trials and associated details. Only interventional trials included.
